# The family life of young people in South Africa from survey data: The case of North-West University

**DOI:** 10.1016/j.dib.2019.104783

**Published:** 2019-11-08

**Authors:** Acheampong Yaw Amoateng, Olusegun Sunday Ewemooje, Elizabeth Biney

**Affiliations:** a Population and Health Research Entity, Faculty of Humanities, North-West University (Mafikeng Campus), North West, South Africa; b Department of Statistics, Federal University of Technology Akure, Akure, Nigeria

**Keywords:** Living arrangement, Premarital sex, South Africa, Wealth index, Youth

## Abstract

This article presents a comprehensive description of student survey data on family life that was collected at the North-West University, South Africa between 2015 and 2016. Responses were obtained from 835 students in the three campuses of the university with the use of multi-stage sampling (stratified and systematic sampling techniques). Data analysis was carried out using tables, frequencies and percentages. The findings reveal that about one-tenth of the respondents live in richer households, one-fifth live in poorer households, while a significant proportion of more than two-thirds live in middle-income households. Less than half of the sampled respondents live with both parents, while about three-in-ten respondents live with either parent. Also, there is a significant relationship between opinion of engaging in premarital sex by young people and household wealth index.

**Table:** Specifications Table

Subject	Social Sciences, Humanities.
Specific subject area	Population Studies, Family Studies, Family Sociology, Youth Studies, Sample Survey.
Type of data	Tables
How data were acquired	Data were acquired through a survey by administering questionnaires to the respondents. A copy of the questionnaire is attached as a supplementary file.
Data format	Raw Analysed
Parameters for data collection	Data were obtained on sociodemographic information of the respondents. Also, information on the living arrangement, religiosity, religious affiliation, substance use and options on the sexuality of young people were obtained.
Description of data collection	Data were obtained from 835 students in the three campuses of North-West University with the use of multi-stage sampling (stratified and systematic sampling techniques).
Data source location	North-West University Mafikeng, Vaal Triangle, Potchefstroom, North West, South Africa
Data accessibility	Data is included in this article

**Table:** 

**Value of the Data** • The data can be used to make inferences about the influence of the family on young people's perceptions and/or behaviours [1]. • The data provide information for researchers in the area of family sociology, youth studies and social sciences broadly. • The data can be used to understand the impact of living arrangements on the lives or development of young people in South Africa [2]. • The data contain items on both the religious affiliation and religiosity of respondents and their family that can be used to make inferences about the role of religion and spirituality in the development or wellbeing of young people in South Africa. • The data can be used to elucidate young people's perceptions of substance use and premarital sex, as well as the prevalence of these issues among the youth population.

## 1 Data

The distribution of respondents by age show that 167 (21.3%) are less than 20 years, 564 (72%) are between 20 and 24 years old, while 52 (6.6%) are 25 years and above (Table 1). This indicates that almost three-quarters of the respondents are emerging adults (20–24 years). 371 (44.4%) of the respondents are male, while 464 (55.6%) are female. The majority (635; 86.6%) of the respondents are Christians and 737 (88.3%) are Black Africans. The household wealth indices of the respondents show that only about one-tenth (10.4%) live in richer households, 166 (19.9%) live in poorer households, while a significant proportion (582; 69.7%) live in middle-income households. Less than half (404; 49%) of the respondents live with both parents in agreement with earlier work of Sibanda [2], 236 (28.6%) live with only a mother, 21 (2.5%) live with only a father, while 79 (9.6%) live with other relatives (aunt, uncle, grandparents). Mafikeng campus has the highest number of respondents of 362 (43.4%), followed by Vaal Triangle campus with 260 (31.1%), while the least is recorded for Potchefstroom 213 (25.5%). Slightly more than half (422; 50.5%) of the respondents had 60–69% as their average mark for the semester, with only 47 (5.6%) having 75% and above (Table 1).

**Table 1:** Classification of the respondents by sociodemographic characteristics.

	Frequency	Valid Percent
**Age**		
<20 years	167	21.3
20–24 years	564	72.0
25 years and above	52	6.6
Total	783	100.0
**Gender**		
Male	371	44.4
Female	464	55.6
Total	835	100.0
**Religious Affiliation**		
Christian	635	86.6
Muslim	11	1.5
Traditional African	56	7.6
Others	31	4.2
Total	733	100.0
**Race**		
Black African	737	88.3
White	62	7.4
Coloured	30	3.6
Indian/Asian	6	.7
Total	835	100.0
**Nationality**		
South Africa	818	98.0
Other SADC countries	15	1.8
Rest of Africa	2	.2
Total	835	100.0
**Household Wealth Index**		
Poorer	166	19.9
Middle	582	69.7
Richer	87	10.4
Total	835	100.0
**Living Arrangement (Living with)**		
Both parent	404	49.0
Only my mother	236	28.6
My mother and stepfather	26	3.2
Only my father	21	2.5
My father and stepmother	10	1.2
Some of the time in my mother's home, and some in my father's home	14	1.7
Other relatives (aunt, uncle, grandparent)	79	9.6
Guardian/foster parent who is not a relative	15	1.8
No parents or guardians (I live alone)	19	2.3
Total	824	100.0
**Campus**		
Mafikeng	362	43.4
Potchefstroom	213	25.5
Vaal Triangle	260	31.1
Total	835	100.0
**Average Mark by Semester**		
Below 50%	9	1.0
50%–59%	182	21.8
60%–69%	422	50.5
70%–74%	175	21.0
75% or more	47	5.6
Total	835	100.0

Table 2 shows that the opinion of engaging in premarital sex by young people is significantly influenced by respondents' household wealth index. Hence, there is a significant relationship between opinion of engaging in premarital sex by young people and household wealth index [3]. Also, it is shown that those from the middle wealth index have a higher percentage, irrespective of respondents' opinion about young people engaging in premarital sex. The least percentage for those who either strongly agreed or agreed is recorded for richer households while respondents who strongly disagreed or disagreed with young people engaging in premarital sex is also recorded for richer households.

**Table 2:** The relationship between household wealth index and opinion of engagement in premarital sex by young people.

	Household Wealth Index			Total	p-value
	Poorer	Middle	Richer		
Strongly agree	20 (22.2%)	61 (67.8%)	9 (10.0%)	90	0.045
Agree	35 (28.0%)	77 (61.6%)	13 (10.4%)	82	
Neutral	46 (15.2%)	229 (75.8%)	27 (9.0%)	302	
Disagree	18 (22.0%)	50 (60.0%)	14 (17.0%)	125	
Strongly disagree	42 (19.2%)	153 (69.8%)	24 (11.0%)	219	

Respondents' academic performance is not significantly influenced by their living arrangement as shown in Table 3, this is supported by earlier works of Azumah et al. [4] and Rabgay [5]. Therefore, there is no significant relationship between student performance at school and whom they live with. However, respondents who live with their both parents are more frequently reported scoring between 70 and 74% as their average semester grade, while those who live with their mother and stepfathers are overrepresented among those failing (i.e. scoring below 50% in average semester grade).

**Table 3:** The relationship between respondents' living arrangement and average mark by semester.

Living Arrangement	Average Mark by semester					Total	p-value
	Below 50%	50%–59%	60%–69%	70%–74%	75% or more		
Both parents	4 (44.4%)	76 (42.0%)	208 (50.1%)	93 (53.8%)	23 (50.0%)	404	0.505
Only my mother	3 (33.3%)	68 (37.6%)	113 (27.2%)	41 (23.7%)	11 (23.9%)	236	
My mother and stepfather	1 (11.1%)	3 (1.7%)	14 (3.4%)	6 (3.5%)	2 (4.3%)	26	
Only my father	0 (0.0%)	10 (5.5%)	7 (1.7%)	3 (1.7%)	1 (2.2%)	21	
My father and stepmother	0 (0.0%)	2 (1.1%)	5 (1.2%)	3 (1.7%)	0 (0.0%)	10	
Some of the time in my mother's home, and some in my father's home	0 (0.0%)	5 (2.8%)	7 (1.7%)	2 (1.2%)	0 (0.0%)	14	
Other relatives (aunt, uncle, grandparent)	1 (11.1%)	12 (6.6%)	42 (10.1)	18 (10.4%)	6 (13.0%)	79	
Guardian/foster parent who is not a relative	0 (0.0%)	1 (0.6%)	9 (2.2%)	4 (2.3%)	1 (2.2%)	15	
No parents or guardians (I live alone)	0 (0.0%)	4 (2.2%)	10 (2.4%)	3 (1.7%)	2 (4.3%)	19	
Total	9	181	415	173	46	824	

Table 4 shows that respondents' living arrangement is significantly influenced by their race. Therefore, there is a significant relationship between the living arrangement and race as earlier shown by Amoateng and Setlalentoa [6]. It shows that Indian/Asian respondents are more likely to live with both parents than other population groups. Black African respondents are more likely to live with either their mothers only or other relatives. White respondents are more likely to live with their fathers and stepmothers, while Coloured respondents are more likely to live with their mothers and stepfathers.

**Table 4:** The relationship between living arrangement and respondents' race.

Living Arrangement	Participants' race				Total	p-value
	Black African	White	Coloured	Indian/Asian		
Both parents	336 (46.2%)	47 (77.0%)	16 (53.3%)	5 (83.3%)	404	0.001
Only my mother	221 (30.4%)	6 (9.8%)	8 (26.7%)	1 (16.7%)	236	
My mother and stepfather	22 (3.0%)	1 (1.6%)	3 (10.0%)	0 (0.0%)	26	
Only my father	20 (2.8%)	1 (1.6%)	0 (0.0%)	0 (0.0%)	21	
My father and stepmother	6 (0.8%)	4 (6.6%)	0 (0.0%)	0 (0.0%)	10	
Some of the time in my mother's home, and some in my father's home	14 (1.9%)	0 (0.0%)	0 (0.0%)	0 (0.0%)	14	
Other relatives (aunt, uncle, grandparent)	75 (10.3%)	1 (1.6%)	3 (10.0%)	0 (0.0%)	79	
Guardian/foster parent who is not a relative	15 (2.1%)	0 (0.0%)	0 (0.0%)	0 (0.0%)	15	
No parents or guardians (I live alone)	18 (2.5%)	1 (1.6%)	0 (0.0%)	0 (0.0%)	19	
Total	727	61	30	6	824	

## 2 Experimental design, materials, and methods

The data comes from the Spirituality, Religion and Positive Youth Development Project, an initiative of Population and Health Research Entity of the North-West University (Mafikeng Campus). Both stratified and multi-stage cluster sampling procedures were employed in selecting respondents from the university. Stratified sampling was used to disaggregate samples from each campus by faculties using population proportional to size based on the population of students by faculties. Teaching departments were grouped into faculties. Therefore, the sampling process began at the faculty level; the student population of each faculty determined the proportion of students for inclusion in the final sample. From each department, core courses at all levels were selected for inclusion. Contact was established with the lecturers of the core courses, thereafter, trained field assistants proceeded to the respective lecture theatres to sample using systematic sampling method with a specific sampling ratio corresponding to class attendance. A sample of 835 students was obtained. Data collection took place between September 2015 and April 2016. A semi-structured questionnaire was used to obtain information on sociodemographic characteristics, religiosity and religious affiliations, substance use and opinion on the sexuality of young people from the respondents. Students also responded to a series of questions relating to their spirituality, academic performance and living arrangements. Since all the questionnaires were anonymous, some students may have been reluctant to respond to some of the items, which led to missing values in some variables, including; age, religious affiliation, living arrangement and premarital sex. However, owing to their theoretical importance and because these missing responses were relatively few and, therefore, negligible, those variables were retained in the dataset and this analysis. The data were then analysed using frequencies, percentages and cross-tabulations.

## 3 Limitations of the data

Some limitations can arise from the data collection method and needs to be taken into consideration when making use of the dataset. First, the sample population consisted of university students which are known to be biased sample for sociological research. Also, social desirability cannot be completely ruled out, particularly in sensitive information regarding sexuality, substance abuse, sexual behaviours and the like.
