# Supplementary material for: The family life of young people in South Africa from survey data: The case of North-West University
Source: Data Brief. 2019 Nov 8;27:104783. doi: 10.1016/j.dib.2019.104783 (PMC6920492; doi:10.1016/j.dib.2019.104783)
Supplement: Multimedia component 1 [file mmc1.docx]

**SPIRITUALITY, RELIGIOSITY AND POSITIVE YOUTH DEVELOPMENT AT THE NORTH-WEST UNIVERSITY, SOUTH AFRICA**

**NORTH-WEST UNIVERSITY**

**APRIL 2015**

| Respondent’s name | |  | | | | | | | | | | | | Interview time |
| --- | --- | --- | --- | --- | --- | --- | --- | --- | --- | --- | --- | --- | --- | --- |
| Respondent’s cell number | |  |  | |  |  |  |  |  | |  |  |  |  |
| Interviewer number |  |  | | Checked | |  | | Back checked | |  | | | | Date |

1. **How old are you? ***(Record age in complete years)*:**
2. **Are you male or female?**

| Male | 1 | Female | 2 |
| --- | --- | --- | --- |

1. **At which type of school did you matriculate? ***Single response***

| Government school in urban area (suburb) | 1 |
| --- | --- |
| Government school in urban area (township) | 2 |
| Government school in rural area (town) | 3 |
| Government school in urban area (township) | 4 |
| Home schooling | 5 |
| Private school | 6 |
| Other (please specify) | 7 |

1. **Which of the following best describe the high school you attended? ***Single response***

| Mainly Black | 1 |
| --- | --- |
| Mainly White | 2 |
| Mainly coloured | 3 |
| Mainly Indian/Asian | 4 |
| Mixed Black/ White/ coloured /Indian/Asian | 5 |

1. **On which campus are you registered as a student? ***Single response***

| Mafikeng Campus | 1 |
| --- | --- |
| Potchefstroom Campus | 2 |
| Vaal Triangle Campus | 3 |

1. *****Record participant’s race – if unsure ask***

| Black African | 1 |
| --- | --- |
| White | 2 |
| Coloured | 3 |
| Indian/Asian | 4 |
| Other: (specify)................................... | 5 |

1. **What is your nationality? ***Single response***

| South Africa | 1 |
| --- | --- |
| Other SADC countries (Namibia, Botswana, Lesotho, Swaziland, Zimbabwe, Zambia, Mozambique, Mauritius, Malawi, Angola, Tanzania, DRC, Madagascar, Seychelles) | 2 |
| Rest of Africa | 3 |
| Other: Specify | 4 |

1. **What is your academic year of study? ***Single response***

| First year | 1 |
| --- | --- |
| Second year | 2 |
| Third or fourth year | 3 |
| Postgraduate degree/diploma | 4 |

1. **What was your first year of registration for the above qualification?**

| 2 | 0 |  |  |
| --- | --- | --- | --- |

1. **In which College are you registered as a student? ***Single response***

| Human and Social Sciences/Arts/Theology/ Humanities | 1 |
| --- | --- |
| Commerce/Economics & Management Sciences/IT/Accounting | 2 |
| Education/ Education Sciences | 3 |
| Engineering | 4 |
| Science/Natural Sciences/Health Science | 5 |
| Agriculture | 6 |
| Law | 7 |

1. **Which of the following applies to you? In the semester the AVERAGE mark for ALL my subjects was... ***Single response***

| Below 40% | 1 |
| --- | --- |
| 40%-49% | 2 |
| 50%-59% | 3 |
| 60%-69% | 4 |
| 70%-74% | 5 |
| 75% or more | 6 |

**12. What language do you (MOSTLY) PREDOMINANTLY SPEAK at home? ONE ANSWER ONLY**

| English | 1 |
| --- | --- |
| Afrikaan | 2 |
| Nguni | 3 |
| North/South Sotho | 4 |
| Non-Nguni | 5 |
| Other African Language specify: | 6 |
| Other European or non-African language specify: | 7 |

**13. Please indicate the highest level of your parents’/guardians’ education**

|  | Father/ Guardian | Mother/ Guardian |
| --- | --- | --- |
| None/ No education | 1 | 1 |
| Primary education (some or complete) | 2 | 2 |
| Some secondary education but not completed | 3 | 3 |
| Matric (Grade 12) | 4 | 4 |
| A college diploma | 5 | 5 |
| A undergraduate degree | 6 | 6 |
| A postgraduate degree | 7 | 7 |

**14. At home, which parents or guardians do you live with?**

| Both my mother and father in the same household | 1 |
| --- | --- |
| Only my mother | 2 |
| My mother and stepfather | 3 |
| Only my father | 4 |
| My father and stepmother | 5 |
| Some of the time in my mother’s home and some in my father’s home | 6 |
| Other relatives (aunt, uncle, grandparent) | 7 |
| Guardian/foster parent who is not a relative | 8 |
| No parents or guardians (I live alone) | 9 |

**15. How would you describe your family’s socioeconomic status compared to other families in the area where you live?**

| We are a lot poorer than most | 1 |
| --- | --- |
| We are a little poorer than most | 2 |
| We have about the same amount of money as most | 3 |
| We are a little richer than most | 4 |
| We are a lot richer than most | 5 |

**16. What is your family’s religious affiliation? ***Single response***

|  | Self | Mother/Guardian | Fathe/Guardianr |
| --- | --- | --- | --- |
| Christian (Protestant) | 1 | 1 | 1 |
| Christian (Catholic) | 2 | 2 | 2 |
| Muslim | 3 | 3 | 3 |
| Judaism | 4 | 4 | 4 |
| Hinduism | 5 | 5 | 5 |
| Traditional African | 6 | 6 | 6 |
| No religious affiliation | 7 | 7 | 7 |
| Other: Specify (specify)......................................... | 8 | 8 | 8 |

**17. If affiliation is Protestant, which Denomination?**

|  | Self | Mother | Father | Guardian |
| --- | --- | --- | --- | --- |
| Anglican | 01 | 01 | 01 | 01 |
| Lutheran | 02 | 02 | 02 | 02 |
| Methodist | 03 | 03 | 03 | 03 |
| Presbyterian | 04 | 04 | 04 | 04 |
| Baptist | 05 | 05 | 05 | 05 |
| Quaker/Friends | 06 | 06 | 06 | 06 |
| Mennonite | 07 | 07 | 07 | 07 |
| Dutch Reformed | 08 | 08 | 08 | 08 |
| Calvinist | 09 | 09 | 09 | 09 |
| Evangelical | 10 | 10 | 10 | 10 |
| Pentecostal | 11 | 11 | 11 | 11 |
| Independent | 12 | 12 | 12 | 12 |
| Church of Christ | 13 | 13 | 13 | 13 |
| Zionist Christian Church | 14 | 14 | 14 | 14 |
| Jehovah’s Witness | 15 | 15 | 15 | 15 |
| Seventh Day Adventist | 16 | 16 | 16 | 16 |
| Mormon | 17 | 17 | 17 | 17 |

**18. How often do you attend church/mosque/synagogue/temple?**

| Never | 0 |
| --- | --- |
| Only on special occasions | 1 |
| Occasionally (several times a year) | 2 |
| Frequent attendance | 3 |
| Fairly Regular (Almost weekly) | 4 |
| Regular (Weekly) | 6 |

**19. How often does your family members (Father/Mother/siblings attend church / mosque / synagogue / temple etc.?**

| Never | 0 |
| --- | --- |
| Only on special occasions | 1 |
| Occasionally (several times a year) | 2 |
| Frequent attendance | 3 |
| Fairly Regular (Almost weekly) | 4 |
| Regular (Weekly) | 6 |

**20. How religious do you consider yourself to be?**

| Not religious at all | 1 |
| --- | --- |
| Somewhat religious | 2 |
| Moderately religious | 3 |
| Very religious | 4 |
| Extremely religious | 5 |

Answer the following questions while taking into consideration your religious beliefs and convictions.

**21. Have you ever used any of the following substances?**

| Marijuana | 1. Yes | 2. No |
| --- | --- | --- |
| Mandrax | 1. Yes | 2. No |
| LSD | 1. Yes | 2. No |
| Cocaine | 1. Yes | 2. No |
| Crack | 1. Yes | 2. No |
| Heroine | 1. Yes | 2. No |
| CAT | 1. Yes | 2. No |
| Ecstasy | 1. Yes | 2. No |
| Inhalants | 1. Yes | 2. No |
| Smoked cigarettes | 1. Yes | 2. No |
| Drunk any alcoholic beverages | 1. Yes | 2. No |

**22. Do you engage in any of the following behaviours currently?**

|  | Never | Sometimes | Always |
| --- | --- | --- | --- |
| Smoking cigarettes | 1 | 2 | 3 |
| Using illicit entertainment drugs such as marijuana | 1 | 2 | 3 |
| Drinking alcohol | 1 | 2 | 3 |
| Engage in violence against other people | 1 | 2 | 3 |

**23. How do you feel about the following statements?**

|  | Strongly agree | Disagree | Neutral | Agree | Strongly disagree |
| --- | --- | --- | --- | --- | --- |
| It is okay for young people to engage in premarital sex. | 1 | 2 | 3 | 4 | 5 |
| It is okay for young people to engage in premarital childbearing. | 1 | 2 | 3 | 4 | 5 |
| It is okay for young people to use drugs. | 1 | 2 | 3 | 4 | 5 |
| It is okay for young people to consume alcohol. | 1 | 2 | 3 | 4 | 5 |
| It is okay for young people to engage in violence for any reason | 1 | 2 | 3 | 4 | 5 |

**THANK YOU FOR YOUR COOPERATION**
